# Contact Lens Associated Bacterial Keratitis: Common Organisms, Antibiotic Therapy, and Global Resistance Trends: A Systematic Review

**DOI:** 10.3389/fopht.2021.759271

**Published:** 2021-12-01

**Authors:** Hossein Hatami, Amir Ghaffari Jolfayi, Ali Ebrahimi, Saeid Golmohammadi, Moein Zangiabadian, Mohammad Javad Nasiri

**Affiliations:** ^1^ Department of Public Health, School of Public Health and Environmental and Occupational Hazard Control Research Center, Shahid Beheshti University of Medical Sciences, Tehran, Iran; ^2^ Student Research Committee, School of Medicine, Shahid Beheshti University of Medical Sciences, Tehran, Iran; ^3^ Department of Microbiology, School of Medicine, Shahid Beheshti University of Medical Sciences, Tehran, Iran

**Keywords:** contact lens, bacterial keratitis, antibiotic therapy, sensitivity, resistance

## Abstract

**Introduction:**

Contact lens wearing has been increased globally during recent decades, which is one of the main risk factors for developing microbial keratitis. Microbial keratitis is a severe and dangerous condition that causes cornea inflammation. It can lead to corneal scarring and perforation or even endophthalmitis and visual loss if it remains untreated. Among bacterial, fungal, protozoal, and viral agents which can cause microbial keratitis, bacteria are the most common cause. Therefore, in this study, we aim to find common causative bacteria, sensitivity, and resistance to antibiotics and the outcome of antibiotic therapy in contact lens-related bacterial keratitis.

**Methods:**

A systematic search was carried out in PubMed/Medline, EMBASE, and Web of Science for published studies and medRxiv for preprints up to February 30, 2021, and May 14, 2021, respectively. A combination of the following keywords was used: “Infection”, “Corneal infection”, “Keratitis”, “Microbial keratitis”, and “Contact lens”, Also, we used the “Contact lenses” MeSH term. Lists of references for each selected article and relevant review articles were hand-searched to identify further studies.

**Results:**

Twenty-six articles were included. From 1991 to 2018, 2,916 episodes of contact lens-related microbial keratitis) CLMK(with 1,642 episodes of proven bacterial keratitis have been reviewed in these studies. Studies were conducted in 17 countries with different geographical regions, and four studies were conducted in Iran, which is the highest number of studies among these countries. According to 20 studies, the mean age of patients was 30.77 years. Females with 61.87% were more than males in 19 studies. A percentage of 92.3% of patients used soft contact lenses, and 7.7% of patients used hard contact lenses (including RGP), according to 16 studies. *Pseudomonas aeruginosa*, *Staphylococcus* spp., and *Serratia marcescens* were the three most common bacteria isolated from samples of patients with contact lens-related bacterial keratitis. Overall, isolated bacteria were most sensitive to fluoroquinolones and aminoglycosides, especially ciprofloxacin and gentamicin respectively, and most resistant against penicillin and cephalosporins especially cefazolin and chloramphenicol. Almost all patients responded well to antibiotic therapy, with some exceptions that needed further surgical interventions.

**Conclusion:**

Antibiotics are efficient for treating almost all patients with contact lens-related bacterial keratitis if they are appropriately chosen based on common germs in every geographical region and the sensitivity and resistance of these germs against them. In this regard, *Pseudomonas aeruginosa* is the most common causative germ of contact lens-associated bacterial keratitis all over the world and is almost fully sensitive to ciprofloxacin. Because of some different results about the sensitivity and resistance of germs against some antibiotics like gentamicin, vancomycin, and chloramphenicol in the Middle East region, especially Iran, more *in vitro* and clinical studies are suggested.

## Introduction

During the recent decades, contact lens wearing has been increased globally from approximately 32 million in 2002 to 40.9 million adult (>18 years old) wearers in 2014 only in the USA, and this number was 140 million worldwide (1, 2). The contact lens global market is estimated at 19.45 billion US dollars in 2024 (3). There are many types of contact lenses available for therapeutic and non-therapeutic purposes; soft and hard or rigid gas permeable (RGB) are two main types (4). Although modern contact lenses are safer than old ones, adverse events like corneal edema due to hypoxia, corneal abrasion, neovascularization, conjunctivitis, midday fogging, inflammation, and infection may occur (5). Contact lens wearing is a prevalent risk factor for microbial keratitis (MK), with an incident rate of approximately 2-20 cases per 10,000 wearers each year (6, 7). Various factors can increase the risk of contact lens-related microbial keratitis like professional occupation compared with being a student, discarding lenses yearly versus fewer periods, showering daily with wearing lenses versus never showering in lenses, and sleeping in lenses (8, 9). Procrastination of therapy results in corneal scarring and perforation, then maybe endophthalmitis and visual loss (10). MK could occur *via* bacterial, fungal, protozoal, and viral agents (11).

Bacterial keratitis is the most common cause of MK, which accounts for about 90% of cases (12); manifestation of bacterial keratitis includes eye discomfort and redness, eyelid swelling, decreased sight, and photophobia (10). Correct identification of the causative pathogen and its virulence factors and using appropriate antibiotics can reduce extended and drastic treatment and avoid further antibiotic resistance. It is also associated with better outcomes and decreased surgical interventions (13).

This systematic review aimed to find common causative germs, sensitivity and resistance to antibiotics, and antibiotic therapy outcomes in contact lens-related bacterial keratitis.

## Methods

This systematic review was conducted according to the “Preferred Reporting Items for Systematic Reviews and Meta-analyses” (PRISMA) statement (14).

### Search Strategy

A systematic search was carried out in the literature from the following bibliographical databases: PubMed/Medline, EMBASE, and Web of Science for published studies and medRxiv for preprints up to February 30, 2021, and May 14, 2021, respectively. Keyword searches were done with combinations of the terms “infection”, “corneal infection”, “keratitis”, “microbial keratitis”, and “contact lens”. Also, we used the “contact lenses” MeSH term. Lists of references of selected articles and relevant review articles were hand-searched to identify further studies. There was no restriction on publication date, but only studies written in English were selected.

### Study Selection

All potentially relevant English articles were screened in two stages for eligibility. Two reviewers independently reviewed titles and abstracts in the first stage. Study types that were included in this stage were clinical retrospective or prospective reviews of patients, case series, and cross-sectional studies that were about infectious keratitis and fit the full-text evaluation criteria. Review articles and case reports were excluded. Discrepancies at this step were discussed with a third reviewer. In the second assessment stage with full-text evaluation, we included studies that discussed contact lens-related bacterial keratitis and examined the sensitivity, resistance, and outcome of antibiotic therapies on common bacterial germs. Therefore, studies that had discussed about other etiological microorganisms such as amoebic, fungal, or viral agents or had focused on infectious keratitis due to factors other than contact lens such as traumatic or post-surgical keratitis were excluded. Other exclusion criteria were studies that discussed about rare causative bacteria, molecular or animal studies, and studies that discussed the protective use of antibiotics as antimicrobial solutions. Disagreements and technical uncertainties were discussed and resolved between review authors.

### Data Extraction

The following variables were extracted from all included studies: first author, study interval, type of study, countries where the study was conducted, study population, number of patients with proven bacterial keratitis, sex and mean age of patients, contact lens regime, diagnostic microbiological tests for bacterial keratitis, and three common isolated bacteria. The three most common sensitive antibiotics and the most resistant ones, and the outcome of antibiotic therapy, were extracted from some studies. Two authors independently extracted the data from the selected studies. The data were jointly reconciled, and disagreements were discussed and resolved between review authors.

## Results

**Figure 1:**
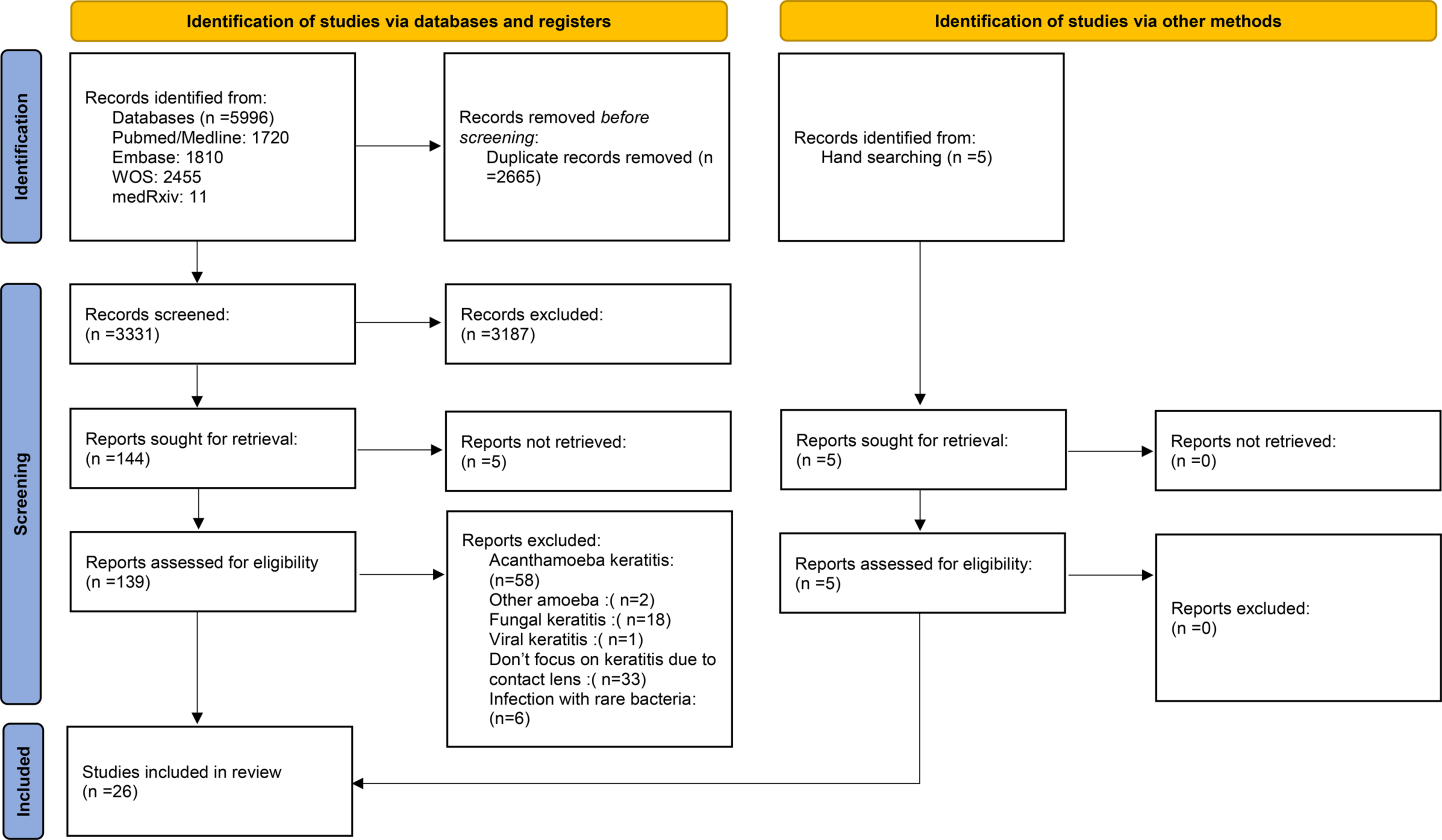
PRISMA 2020 flow diagram for new systematic reviews which included searches of databases, registers, and other sources.

The selection process of articles is shown in **Figure 1**. Twenty-six articles were included and classified into the following: 15 retrospective reviews of patients (15–29), seven prospective reviews of patients (30–36), three cross-sectional studies (37–39), and one case series (40). Four studies have been conducted in Iran; three in India; two studies in the USA, Australia, Netherlands, China; and one study each in Switzerland, Portugal, Pakistan, Thailand, Brazil, Turkey, France, Egypt, UK, Japan, and Belgium (**Table 1**). A total of 2,916 episodes of CLMK with 1,642 episodes of proven bacterial keratitis (isolated or polymicrobial) have been reviewed in studies from 1991 to 2018 (**Tables 1** and **2**). According to 20 studies, the mean age of patients was 30.77 years. Females with 61.87% were more than males in nineteen studies. A percentage of 92.3% of patients used soft contact lenses, and 7.7% of patients used hard contact lenses (including RGP), according to 16 studies (**Table 2**). Diagnostic microbiological tests are shown in **Table 2**.

**Table 1:** Characteristics of all included studies.

Title of article	Author	Year	Country	Type of study
Microbial Analyses of Contact Lens-Associated Microbial Keratitis	Konda et al. (15)	January 2001 to November 2011	India	Retrospective review of patients
Initial treatment of Pseudomonas aeruginosa contact lens-associated keratitis with topical chloramphenicol, and effect on outcome	Bourkiza et al. (16)	2007 to 2009	UK	Retrospective review of patients
Study on Prevalence, Antibiotic Susceptibility, and *tuf* Gene Sequence–Based Genotyping of Species-Level of Coagulase-Negative Staphylococcus Isolated From Keratitis Caused by Using Soft Contact Lenses	Faghri et al. (30)	January 2013 to August 2013	Iran	prospective review of patients
Clinical Presentation and Antibiotic Susceptibility of Contact Lens Associated Microbial Keratitis	Hedayati et al. (37)	June 2012 to June 2013	Iran	cross-sectional
Antibiotic Susceptibility Patterns of Pseudomonas Corneal Ulcers in Contact Lens Wearers	Mohammadpour et al. (38)	March 2009 to March 2010	Iran	cross-sectional
Sensitivity Pattern of Bacteria Isolated from Contact Lens Wearers in the Faculty of Pharmacy, Karachi University Student Population	Rahim et al. (31)	February 2005 to January 2006	Pakistan	prospective review of patients
Clinical presentation and morbidity of contact lens–associated microbial keratitis: a retrospective study	Hoddenbach et al. (17)	January 1, 2005, to December 31, 2009	Netherland	Retrospective review of patients
Contact Lens–Induced Infectious Keratitis in Japan	Inoue et al. (18)	From January 1999 to December 2003	Japan	Retrospective review of patients
Contact lens-related microbial keratitis in Egypt: 5y epidemiological study	Khater et al. (32)	first of January 2009 to the end of December 2013	Egypt	Prospective review of patients
Clinical Presentation and Microbial Analyses of Contact Lens Keratitis; an Epidemiologic Study	Rasoulinejad et al. (39)	2011 to 2013	Iran	cross-sectional
Bacterial keratitis: Predisposing factors, clinical and microbiological review of 300 cases	Bourcier et al. (19)	January 1998 to September 1999	France	Retrospective review of patients
Clinical and microbiological characteristics of corneal ulcers in a Thai referral center	Kampitak et al. (20)	January 2006 and December 2010	Thailand	Retrospective review of patients
Colored cosmetic contact lenses: An unsafe trend in the younger generation	Singh et al. (40)	November 2009 to February 2010	India	Case series
Contact Lens Versus Non-Contact Lens-Related Corneal Ulcers at an Academic Center	Bennett et al. (21)	1999 to 2016	USA	Retrospective review of patients
Contact lens-associated microbial keratitis	Moriyama et al. (22)	January 2002 to December 2007	Brazil	Retrospective review of patients
Contact Lens-Associated Microbial Keratitis in a Tertiary Eye Care Center in Turkey	Karaca et al. (23)	2012 to 2018	Turkey	Retrospective review of patients
Bacterial keratitis: A prospective clinical and microbiological study	Schaefer et al. (33)	March 1, 1997, to November 30, 1998	Switzerland	Prospective review of patients
Clinical and Microbiological Profile of Bacterial Microbial Keratitis in a Portuguese Tertiary Referral Center-Where Are We in 2015?	Ferreira et al. (24)	September 2007 to August 2015	Portugal	Retrospective review of patients
Contact lens-related corneal ulcers requiring hospitalization: A 7-year retrospective study in Belgium	Verhelst et al. (25)	January 1997 to December 2003	Belgium	Retrospective review of patients
Relationship Between Climate, Disease Severity, and Causative Organism for Contact Lens–Associated Microbial Keratitis in Australia	Stapleton et al. (34)	October 1, 2003, and September 30, 2004	Australia	prospective review of patients
Trends in Contact Lens–associated Microbial Keratitis in Southern India	Sharma et al. (26)	February 1991 and September 2000	India	Retrospective review of patients
Incidence of contact-lens-associated microbial keratitis and its related morbidity	Cheng et al. (35)	April 1 and June 30, 1996	Netherlands	prospective review of patients
Microbial Keratitis Profile at a University Hospital in Hong Kong	Lai et al. (27)	January 2010 to June 2012	China	Retrospective review of patients
Incidence and risk factors for microbial keratitis in Hong Kong: comparison with Europe and North America	Lam et al. (36)	April 1997 and August 1998	China	prospective review of patients
Trends in contact lens microbial keratitis 1999 to 2015: a retrospective clinical review	Green et al. (29)	January 1999 to December 2015	Australia	Retrospective review of patients
Visual Outcome, Microbiological Profile and Antibiotic Sensitivity of Infectious Keratitis in a Tertiary Referral Center	Stanfield et al. (28)	January 1, 2014 to December 31, 2018	USA	Retrospective review of patients

UK, United Kingdom; USA, United States of America.

**Table 2:** Epidemiologic and demographic features of studies.

Author	Study population	No. proven bacterial keratitis	Age in years(mean)	Sex	Contact lens types	Microbiological tests
Konda et al. (15)	125 eyes of 123 patients	83 (71%)	24.14 in male 26.7 in female total: 25.38	63 male (51.2%) and 60 female (48.8%)	—	Corneal culture Contact lens culture Contact lens case culture
Bourkiza et al. (16)	139 patients	139 (100%)	28	57 male (41%) and 82 female (59%)	Soft: 121 (87%) RGP: 4 (2.8%) Cosmetic: 2 (1.4%) Therapeutic: 6 (4.3%) Not recorded: 6 (4.3%)	Corneal cultures with Gram-negative bacterial isolates
Faghri et al. (30)	77 patients	60 (80%)	26	23 male (29.8%) and 54 female (70.2%)	Soft: 77 (100%)	Corneal culture *tuf* gene sequencing
Hedayati et al. (37)	33 eyes of 26 patients	25 (75.7%)	23.88	2 male (7.7%) and 24 female (92.3%)	Soft: 26 (100%)	Corneal scraping culture
Mohammadpour et al. (38)	52 patients	52 (100%)	21.5	9 male (17.3%) and 43 female (82.7%)	Soft: 52 (100%)	Perform smears for gram staining and then culture the specimens
Rahim et al. (31)	100 patients	100 (100%)	—	—	—	Culture of bacterial isolates from conjunctiva
Hoddenbach et al. (17)	109 patients	—	33.3	Male: 42.2% (46) Female: 57.8% (63)	Soft: 97 (88.9%) RGP: 12 (11.1%)	Corneal culture Contact lens culture contact lens box culture
Inoue et al. (18)	67 eyes of 66 patients	—	37	Male-to-female ratio was 1:0.91 35 male (52.35%) and 31 female (47.65%)	Soft: 48 (71.6%) Hard (including RGP): 19 (22.4%)	Culture of corneal scrapings or eye discharge or contact lens or contact lens preservative solution
Khater et al. (32)	151 patients	Only bacteria 43 (28.5%) Mixed bacteria and fungi 53 (35.1%)	31	18 male (11.9%) and 133 female (88.1%)	—	Culture of storage cases solutions or the contact lens itself besides corneal swabs or biopsy
Rasoulinejad et al. (39)	17 eyes of 14 patients	14 (82.3%)	21.58	14 female (100%)	Soft: 14 (100%)	Corneal culture
Bourcier et al. (19)	151 eyes	95 (62.9%)	32	—	Soft: 135 (89.4%( RGP: 13 (8.6%) Hard PMMA: 3 (2%)	Corneal culture Contact lens culture Storage cases culture
Kampitak et al. (20)	35 patients	10 (29%)	25.6	5 (14.3%) male and 30 female (85.7%)	—	Corneal culture
Singh et al. (40)	13 patients	12 (92.3%)	19	8 male (61.5%) and 5 female (38.5%)	—	Corneal culture
Bennett et al. (21)	319 patients	116 (36.3%)	32.7	121 male (38%) and 198 female (62%)	—	Corneal culture
Moriyama et al. (22)	239 patients	166 (69.4%)	29.75	Male-to-female ratio 1:1.26 106 male (44.25%) and 133 female (55.75%)	Soft: 96 (88.07%) RGP: 11 (10.09%) Piggy-back lenses: 2 (1.83%)	Corneal culture
Karaca et al. (23)	62 patients	40 (64.5%)	24.5	22 male (35.4%) and 40 female (64.6%)	Soft: 61 (98.4%) RGP: 1 (1.6%)	Corneal culture Contact lens culture Storage cases culture
Schaefer et al. (33)	31 patients	28 (90.3%)	—	—	—	Corneal culture
Ferreira et al. (24)	65 patients	—	36	—	—	Corneal culture
Verhelst et al. (25)	107 patients	72 (67.2%)	28.8	42 male (39.2%) 65 female (60.8%)	Soft: 99 (92.5%) RGP: 8 (7.5%)	Corneal culture Contact lens culture Storage cases culture
Stapleton et al. (34)	236 patients	59 (25%)	—	—	—	Corneal culture
Sharma et al. (26)	28 patients	25 (89.2%)	22.3	12 male (42.8%) 16 female (57.2%)	Soft: 15 (53.5%) RGP: 6 (21.4%) Therapeutic bandage contact lenses: 4 (14.2%) SilSoft lenses: 3 (10.7%)	Corneal culture
Cheng et al. (35)	92 patients	29 (31.5%)	32.6	47 male (51%) 45 female (49%)	Soft: 75 (82%) RGP: 17 (18%)	Corneal culture
Lai et al. (27)	23 patients	14 (60.8%)	27.7	—	Soft: 23 (100%)	Corneal culture
Lam et al. (36)	59 patients	22 isolates (including polymicrobial cultures)	—	20 male (34%) 39 female (66%)	Soft: 58 (98%) RGP: 1 (2%)	Corneal culture
Green et al. (29)	372 CLMK episodes of 324 patients	357 CLBK episodes (96%)	36.47	43.2% male (140) and 56.8% female (184)	Soft: 215 (66.4%) RGP: 6 (1.9%)	Corneal scrape culture
Stanfield et al. (28)	214 eyes	71 (33.1%)	—	—	—	Culturing for a corneal infection

CLMK, contact lens related microbial keratitis; CLBK, contact lens related bacterial keratitis.

### The Most Common Isolated Bacteria

Twenty-four of 26 studies perused the most common isolated bacteria from cultured samples; two of them (16, 38) only had Pseudomonas spp. in their survey. Among these 24 studies, Pseudomonas spp. especially *Pseudomonas aeruginosa* was the most common isolated bacteria from cultured samples (625 episodes among three common germs in studies with raw data) and were the first common bacteria in 18 studies and the second common bacteria in five studies. Only the study by Inoue et al. (18) had not reported Pseudomonas among its three common isolated bacteria. Staphylococcus spp. especially coagulase-negative staphylococcus spp. (CoNS) Staphylococcus epidermidis was the second most common bacteria from cultured samples. In five studies, they were the first common bacteria (438 episodes of Staphylococcus spp. and 364 episodes of coagulase-negative staphylococcus spp. among three common germs in studies with raw data). Serratia spp. was the third common bacteria, and in the study by Cheng et al. (35), *Serratia marcescens* and *Pseudomonas aeruginosa* were the first common bacteria from cultured samples (44 episodes among three common germs in studies with raw data). Ferreira et al. (23) and Verhelst et al. (24) noticed that *Pseudomonas aeruginosa* was significantly associated with a worse clinical manifestation than other causative organisms. The risk of microbial keratitis in extended-wear and also daily-wear soft contact lenses was greater than that in daily-wear RGP lenses according to Cheng et al. (34). Bourcier et al. (18) and Rasoulinejad et al. (38) mentioned that gram-negative bacteria especially *Pseudomonas aeruginosa* were more associated with soft contact lenses, whereas according to Inoue et al. (17), gram-positive bacteria like staphylococcus species were the most common bacteria in soft and hard contact lenses.

The three most common bacteria that were isolated from samples in each study are shown in **Table 3**.

**Table 3:** Three most common bacteria in studies.

Author	The first most common bacteria	The second most common bacteria	The third most common bacteria
Konda et al. (15)	*Pseudomonas* spp. 61 (73.5%)	*Staphylococcus epidermidis* 4 (4.8%) *Serratia* spp. 4 (4.8%)	*Other coagulase-negative staphylococci* 2 (2.4%)
Bourkiza et al. (16)	139 Pseudomonas spp. among 149 cases with culture-proven Gram-negative organisms	10 Serratia spp. among 149 cases with culture-proven Gram-negative organisms	—
Faghri et al. (30)	Coagulase-negative staphylococcus 38 (49.3%) (S. epidermidis: 31)	Pseudomonas aeruginosa 5 (7%) Enterobacter aerogenes 5 (7%)	Micrococcus luteus 3 (3.9%) Bacillus spp. 3 (3.9%) Serratia spp. 3 (3.9%) Klebsiella spp. 3 (3.9%)
Hedayati et al. (37)	Pseudomonas aeruginosa 20 (80%)	Staphylococcus aureus 3 (12%)	Enterobacter 2 (8%)
Mohammadpour et al. (38)	Pseudomonas aeruginosa 52 (100%)	—	—
Rahim et al. (31)	S. epidermidis 41 (41%)	Pseudomonas aeruginosa 39 (39%)	S. aureus 11 (11%)
Hoddenbach et al. (17)	Pseudomonas aeruginosa 68.8%	Serratia spp.	Stenotrophomonas maltophilia
Inoue et al. (18)	Staphylococcus epidermidis 13 (36.1%)	Staphylococcus aureus 5 (14.3%)	Corynebacterium spp. 4 (11.4%)
Khater et al. (32)	Gram positive bacteria (S. aureus, S. epidermidis, pneumococci) 27 (63%)	Gram-negative bacteria (Pseudomonas aeruginosa) 16 (37%)	—
Rasoulinejad et al. (39)	Pseudomonas aeruginosa 11 (78.6%)	Staphylococcus aureus 2 (14.3%)	Enterobacter 1 (7.1%)
Bourcier et al. (19)	Coagulase negative staphylococcus 47 (49.4%)	Pseudomonas aeruginosa 18 (18.9%)	Propionibacterium acnes 14 (14.7%)
Kampitak et al. (20)	Pseudomonas aeruginosa 9 (90%)	Acinetobacter baumannii 1 (10%)	—
Singh et al. (40)	Pseudomonas aeruginosa 7 (54%)	Staphylococcus aureus 3 (25%)	Staphylococcus epidermidis 2 (17%)
Bennett et al.	Pseudomonas spp. 62 (53.4%)	Coagulase negative staphylococcus 17 (14.6%)	Staphylococcus aureus 15 (12.9%)
Moriyama et al. (22)	Coagulase negative staphylococcus 74 (44.5%)	Pseudomonas spp. 32 (19.2%)	Corynebacterium spp. 20 (12%)
Karaca et al. (23)	Pseudomonas aeruginosa 17 (42.5%)	Serratia marcescens 8 (20%)	Stenotrophomonas maltophilia 5 (12.5%)
Schaefer et al. (33)	Gram negative bacteria 12 (43%) mostly *Pseudomonas* species	—	—
Ferreira et al. (24)	Pseudomonas aeruginosa 36.3%	Serratia spp. 18%	S. epidermidis and other coagulase-negative Staphylococcus 13.6%
Verhelst et al. (25)	Pseudomonas aeruginosa 54 (75%)	Serratia marcescens 16 (22.2%)	Klebsiella oxytoca 10 (13.8%)
Stapleton et al. (34)	*Pseudomonas* spp. 35 (59.3%)	Serratia spp. 6 (10.1%)	Staphylococcus aureus 4 (6.7%) Coagulase-negative staphylococcus 4 (6.7%)
Sharma et al. (26)	*Pseudomonas* spp. *13 (52%)*	Staphylococcus spp. 6 (24%)	Streptococcus spp. 4 (16%)
Cheng et al. (35)	*Pseudomonas aeruginosa 7 (24.1%)* *Serratia marcescens 7 (24.1%)*	Staphylococcus spp. 6 (20.6%)	Enterobacter spp. 5 (17.2%)
Lai et al. (27)	*Pseudomonas aeruginosa 13 (92.8%)*	—	—
Lam et al. (36)	*Pseudomonas aeruginosa 12 (54.5%)*	*Gram-positive bacteria 5* (22.7%) Gram-negative bacteria other than P. aeruginosa 5 (22.7%)	—
Green et al. (29)	Pseudomonas aeruginosa 181 (50.70%)	Coagulase-negative Staphylococci 111 (31%)	Staphylococcus aureus (non-MRSA) 10 (2.8%)
Stanfield et al. (28)	Pseudomonas aeruginosa 29 (40.8%)	Staphylococcus epidermidis 11 (15.5%)	Staphylococcus aureus 9 (12.6%)

### Sensitivity and Resistance to Antibiotics

Fifteen studies reported the sensitivity and resistance of isolated bacteria to antibiotics. Eight studies reported the sensitivity and resistance of each bacteria separately. In the remaining seven studies, only the overall sensitivity and resistance of discussed bacteria to antibiotics were reported. In studies by Mohammadpour et al. (38) and Bourkiza et al. (16) which only discussed the treatment of Pseudomonas spp., isolated bacteria were 100% sensitive to fluoroquinolones, especially ciprofloxacin. Also, isolated bacteria in the study by Mohammadpour et al. (38) were 100% sensitive to ceftazidime. Green et al. (29) reported that none of the cultured isolates of *P. aeruginosa* was resistant to fluoroquinolones. Lai et al. (27) and Moriyama et al. (22) noticed that cultured *Pseudomonas aeruginosa* samples were 100% sensitive to ciprofloxacin and gentamicin in the former study and ciprofloxacin, ofloxacin, gatifloxacin, amikacin, and tobramycin in the latter. Hoddenbach et al. (17) showed that *P. aeruginosa* is sensitive to ofloxacin and gentamicin by 98.7% and 97.3%, respectively, and Bennett et al. (21) noticed that *P. aeruginosa* is 100% sensitive to oxacillin. On the other hand, Rahim et al. (31) represented that *P. aeruginosa* had 82% sensitivity to ciprofloxacin and was most sensitive to imipenem with 84.4% sensitivity. In the study by Singh et al. (40), P. aeruginosa had only 57.1% and 14.2% sensitivity to other fluoroquinolones like moxifloxacin and levofloxacin, respectively. According to studies by Hedayati et al. (37) and Rasoulinejad et al. (39), P. aeruginosa is 100% resistant to gentamicin. Hoddenbach et al. (17) noticed that resistance to cefazolin in P. aeruginosa is 99.3%. Isolated P. aeruginosa in the study by Bourkiza et al. (16) showed 100% resistance to chloramphenicol, but Mohammadpour et al. (38) showed 100% resistance for cefazolin and vancomycin and 97% for chloramphenicol.

*S. epidermidis* and *S. aureus*, according to Rahim et al. (31), had the most sensitivity to imipenem with 100% sensitivity. In this study, ciprofloxacin is in second place in which S. aureus showed 100% sensitivity and S. epidermidis 92.9% sensitivity to it. Moriyama et al. (22) represented that coagulase-negative *staphylococcus* spp. had 100% sensitivity to ciprofloxacin, ofloxacin, amikacin, tobramycin, gentamicin, cephalothin, and oxacillin. S. aureus and CoNS in the study by Bennett et al. (21) showed 87% and 83% sensitivity to oxacillin, respectively. Conversely, Singh et al. (40) reported that the S. aureus sensitivity to moxifloxacin, levofloxacin, and amikacin was 33.3%, and S. epidermidis was 50% sensitive to moxifloxacin. In the study by Hedayati et al. (37), all three S. aureus samples were resistant to ciprofloxacin but were sensitive to gentamicin.

Hoddenbach et al. (17) showed that Serratia spp. were 100% sensitive to gentamicin and 90% sensitive to ofloxacin but were 90% resistant to cefazolin. Also, Karaca et al. (23) reported that both Serratia marcescens and P. aeruginosa were 100% sensitive to vancomycin and ceftazidime.

Among studies that reported the overall sensitivity and resistance of all discussed bacteria (gram-negative and gram-positive bacteria like P. aeruginosa, Serratia spp., S. epidermidis, S. aureus, etc.) to antibiotics, bacteria were most sensitive to ciprofloxacin in studies by Hedayati et al. (37) and Rasoulinejad et al. (39) with 86% and 71.4% sensitivity in each study respectively. Also, ciprofloxacin was the second and third antibiotics in the studies by Sharma et al. (26)and Faghri et al. (30) in which bacteria had the most sensitivity to it, with 88% and 86.8%, respectively. In these two studies, gentamicin was the first antibiotic in that order, in which bacteria had 100% sensitivity in the study by Faghri et al. (30) and 92% sensitivity in that of Sharma et al. (26). Other fluoroquinolones were discussed in the study by Konda et al. (15), and gatifloxacin, ofloxacin, and gentamicin were the first three antibiotics that bacteria were most sensitive to, with 89%, 88%, and 86% sensitivity to each of them, respectively. Green et al. (29) reported that *P. aeruginosa, S. epidermidis,* and *S. aureus* had 100% sensitivity to vancomycin. Bacteria had 100% resistance to penicillin in two studies (37, 39) and 71.1% resistance in another study (30). Konda et al. (15) reported that Pseudomonas spp., Serratia spp., and CoNS were most resistant to chloramphenicol, while in the study by Faghri et al. (30), these bacteria had 94.7% sensitivity to chloramphenicol. Bacteria in the study by Green et al. (29) were most resistant against cephalosporins. For example, all bacteria excluding Enterobacter had 100% resistance against cefixime in the study by Rasoulinejad et al. (39) and had 56% resistance against cefazolin in Sharma et al. (26). The three most effective antibiotics and the most ineffective ones are shown in **Table 4**.

**Table 4:** The three most effective and the most ineffective antibiotics.

Author	The first most effective antibiotic	The second most effective antibiotic	The third most effective antibiotic	The most ineffective antibiotic
Konda et al. (15)	Gatifloxacin 89%	Ofloxacin 88%	Gentamicin 87%	Chloramphenicol
Bourkiza et al. (16)	Fluoroquinolone (ciprofloxacin) 100%	—	—	Chloramphenicol 100%
Faghri et al. (30)	Gentamicin 100%	Chloramphenicol 94.7%	Ciprofloxacin 86.8%	Penicillin 71.1%
Hedayati et al. (37)	Ciprofloxacin 86%	Imipenem, meropenem, and ceftazidime 76%	—	Penicillin 100%
Mohammadpour et al. (38)	Ceftazidime and ciprofloxacin 100%	Amikacin 97%	Imipenem 96%	Cefazolin and vancomycin 100%
Rahim et al. (31)	Imipenem 100% for S. epidermidis and S. aureus 84.4% for P. aeruginosa	Ciprofloxacin 100% for S. aureus 92.9% for S. epidermidis 82% for P. aeruginosa	—	Amoxicillin, cephradine, neomycin, and chloramphenicol
Hoddenbach et al. (17)	Ofloxacin 100% for S. maltophilia 98.7 % for P. aeruginosa 90 % for Serratia spp	Gentamicin 100 % for Serratia spp. 97.3 % for P. aeruginosa 57.1% for S. maltophilia	—	Cephazolin 100% for S. maltophilia 99.3 % for P. aeruginosa 90 % for Serratia spp.
Rasoulinejad et al. (39)	Ciprofloxacin, 71.4%	ceftazidime, imipenem, and meropenem		Penicillin 100%
Singh et al. (40)	Moxifloxacin 57.1% for P. aeruginosa 33.3% for S. aureus 50% for S. epidermidis	Amikacin 28.5% for P. aeruginosa 33.3% for S. aureus	Levofloxacin 14.2% for P. aeruginosa 33.3% for S. aureus	—
Bennett et al. (21)	Oxacillin 100% for P. aeruginosa 87% for S. aureus 83% for CoNS	—	—	—
Moriyama et al. (22)	Ciprofloxacin and ofloxacin and amikacin and tobramycin 100% for CoNS and P. aeruginosa	Gatifloxacin 100% for P. aeruginosa 97% for CoNS Gentamicin 100% for CoNS 97% for P. aeruginosa Cephalothin and oxacillin 100% for CoNS	—	—
Karaca et al. (23)	Vancomycin and ceftazidime 100%	—	—	—
Sharma et al. (26)	*Gentamicin* 92%	*Ciprofloxacin* *88%*	*Cefazolin* 44%	—
Lai et al. (27)	*Gentamicin and ciprofloxacin* *100%*	—	—	—
Green et al. (29)	Vancomycin 100%	Fluoroquinolones	Chloramphenicol	Cephalosporins

CoNS, coagulase negative staphylococcus.

### The Outcome of Antibiotic Therapy

The outcome of experimental or antibiogram-guided antimicrobial therapy was discussed in 11 studies. Therapeutic penetrating keratoplasty, therapeutic graft, amniotic membrane transplantation, anterior lamellar keratoplasty, anterior lamellar corneal transplants, RGB lens, and phototherapeutic keratectomy were additional therapies in patients that did not respond to antibiotic therapy and had complications like corneal perforation. Singh et al. (40) represented that treatment outcome was good among all cases with prescribed topical antimicrobials, and none of them have required surgical interventions; also, in the study by Konda et al. (15), approximately all patients were treated with choosing antibiotics *via* antibiogram. Among 61 pseudomonas spp., four of them showed *in vitro* resistance to multiple antibiotics containing aminoglycosides, fluoroquinolones, and third cephalosporins. Three cases showed the relevant clinical results to ciprofloxacin eye drops, and one case needed penetrating keratoplasty due to insufficiency of medical therapy. Mohammadpour et al. (38) observed 81% prosperous clinical response with antibiotic therapy, and Sharma et al. (26) noticed that among 28 patients, ulcers of 24 (85.7%) patients were healed with laboratory-based medical therapy, while other patients needed penetrating keratoplasty. According to the study by Bourkiza et al. (16), experimental use of chloramphenicol against Pseudomonas spp. leads to greater ulcer size and worse visual acuity at presentation to the hospital, more median interval to final follow-up, and more complications like a vascularized scar. However, VA at the final review was not statistically different between chloramphenicol and non-chloramphenicol groups. Hoddenbach et al. (17) reported that three perforating keratoplasties were managed in an emergency setting because of corneal perforation due to *Pseudomonas aeruginosa*. Karaca et al. (23) studied that the mean best-corrected visual acuity (BCVA) increased with antibiotic therapy, and according to culture results, *P. aeruginosa* infections were associated with significantly worse BCVA. In the study by Green et al. (29), only one 68-year-old female had a poor outcome due to cultured MRSA from scraping cornea resistant to multiantibiotics like cephalosporins and fluoroquinolones. Finally, she was treated with topical vancomycin while her visual acuity was hand movements. Twenty-one patients (6.5%) with CLMK required surgical interventions or showed complications in this study. The outcome of antibiotic therapy is shown in **Table 5**.

**Table 5:** Outcome of antibiotic therapy.

Author	Outcome
**Konda et al.** (**15**)	Approximately all patients were treated with choosing antibiotics *via* antibiogram. Among 61 pseudomonas spp., four of them showed *in vitro* resistance to multiple antibiotics containing aminoglycosides, fluoroquinolones, and third cephalosporins. Three cases showed suitable clinical result to ciprofloxacin eye drops. One case needs penetration of keratoplasty due to insufficiency of medical therapy.
**Bourkiza et al.** (**16**)	At presentation to the hospital, the chloramphenicol group had a larger size of ulcer and worse VA than the non-chloramphenicol group, while final VA was not statistically different in the final examination. The average period follow-up of patients for the chloramphenicol group was 37 days versus 21 days for the non-chloramphenicol group. Six complications including 3 vascularized scar and 3 therapeutic graft happen in the chloramphenicol group versus 2 therapeutic graft in the non-chloramphenicol group.
**Hedayati et al.** (**37**)	Among all cases, 57.7% were treated outpatients; 34.6% and 7.7% of them were admitted and need to surgical interventions, respectively. The median treatment interval was 31 ± 6 days in the outpatient case and 84 ± 12 days in the inpatient case. Treatment outcomes were excellent in 24.2%, good in 45.5%, and poor in 30.3% based on results.
**Mohammadpour et al.** (**38**)	39 cases (75%) required hospitalization, while 13 cases (25%) were managed in outpatient and none of the patients needed hospitalization during the follow-up. A prosperous clinical response of 81% was seen with antibiotic therapy. Ten cases (10%) with mean age of 21 years and 4 × 4 mm corneal ulcer on average required amniotic membrane transplantation and 58% of them had hypopyon.
**Hoddenbach et al.** (**17**)	Corneal transplantation was needed for 22 eyes (20.2%), including 17 perforating keratoplasties, 3 deep anterior lamellar keratoplasties, and 2 anterior lamellar corneal transplants. Due to corneal perforation, three patients with perforating keratoplasty were managed in emergency settings and 19 keratoplasties were conducted for culture-positive samples of P. aeruginosa due to major loss of visual acuity because of scarring. None of the cases required evisceration or enucleation. Forty-seven patients (43.1%) needed rigid gas permeable (RGP) lenses for achieving proper vision, whereas 33 patients (30.3%) did not need further interventions like RGP or surgery.
**Rasoulinejad et al. **(**39**)	Outcome of treatment was excellent in 23.5%, good in 47.1%, and poor in 29.4% of patients that required penetrating keratoplasty intervention
**Singh et al.** (**40**)	Treatment outcome was good among all cases with prescribed topical antimicrobials, and none of them required surgical interventions.
**Karaca et al.** (**23**)	There was a significant negative correlation between best-corrected visual acuity (BCVA) and P. aeruginosa keratitis. At the end of follow-up, the mean BCVA was increased from 0.7 log of minimal angle of resolution (logMAR) (0–3) to 0.1 logMAR (0–0.4). Average time for hospitalization was 16.7 days, for complete healing was 16.7 mouth and 6.3 mouth for follow-up.
**Sharma et al.** (**26**)	Among 28 patients, ulcers of 24(85.7%) patients were healed the ulcers with laboratory-based medical therapy, while other patients needed penetrating keratoplasty.
**Lai** **et al.** (**27**)	On presentation, the mean logMAR visual acuity of all patients was 0.99 and was increased to 0.34 and 0.26 at 1 and 3 months after treatment, respectively. Only 37 and 19 cases were recorded at 1 and 3 months, respectively, due to neglecting follow-up or being released from clinic. The mean logMAR visual acuity was improved -0.77 at 3 months in between 19 patients.
**Green et al.** (**29**)	Among all CLMK cases only one 68-year-old female had a poor outcome related to cultured MRSA from scraping cornea that was resistant to multi-antibiotics like cephalosporins and fluoroquinolones. Finally, she was treated with topical vancomycin while her visual acuity was hand movements. 21 patients (6.5%) with CLMK required surgical interventions or showed complications, which included penetrating keratoplasty in 18 (6.1%) patients, phototherapeutic keratectomy in 2 cases, and 2 patients had corneal perforation.

VA, visual acuity; CLMK, contact lens related microbial keratitis.

## Discussion

Among the reviewed articles, the most common isolated bacteria were *Pseudomonas aeruginosa* which was considered separately in the two articles due to its high prevalence and importance of treatment. Although it was not reported as a common germ in some studies, no other bacteria were mentioned besides *P. aeruginosa* in all reviewed studies. The next common organism was *Staphylococcus* spp. especially coagulase-negative spp. such as Staphylococcus epidermidis, and the third organism was Serratia marcescens. In some articles’ rankings, these agents fluctuated, but because of different geographical areas where studies were conducted, somehow variation in the frequency of microorganisms is acceptable and not so far unexpected.

In the treatment approaches, *Pseudomonas* is most considered in the studies, and there is a consensus on notable sensitivity to fluoroquinolones, especially ciprofloxacin as a drug of choice in this issue. Ofloxacin and gatifloxacin are two effective antibiotics against Pseudomonas, but moxifloxacin and levofloxacin which are other members of fluoroquinolones were not effective in this regard; these antibiotics are known as respiratory fluoroquinolones, and their inefficacy as a topical drug is presumable (41).

Mohammadpour et al. (38), Bourkiza et al. (16), Lai et al. (27), and Moriyama et al. (22) mentioned that *P. aeruginosa* was 100% sensitive to ciprofloxacin, but some studies like Rahim et al. (31) have mentioned not complete but 82% sensitivity to this antibiotic. Due to the critical role of ciprofloxacin as a leading antibiotic in the eradication of P. aeruginosa, this slight change in sensitivity should be noticed. According to the date of studies, the study by Rahim et al. (31) is conducted before those of Mohammadpour et al. (38), Bourkiza et al. (16), and Lai et al. (27), so antibiotic resistance development is not probable. We should also consider that the studies by Mohammadpour et al. (38) and Rahim et al. (31) were conducted in two proximal geographical areas respectively in Iran and Pakistan, so we do not expect so many differences between these two studies. This difference may be related to the source of the isolated bacteria in the study by Rahim et al. (31) which was from the conjunctiva, but this hypothesis needs more investigation.

Using ciprofloxacin as an antibiotic for Staphylococcus spp. was confirmed by the study of Rahim et al. (31) in which S. aureus had complete sensitivity to ciprofloxacin, but conversely, in a study held by Hedayati et al. (37), all three S. aureus samples were resistant to ciprofloxacin. It can be supposed that this is due to the small sample size and therefore a sampling bias, but a 100% resistance even in a small sample size is remarkable. The study by Rahim et al. (31) was held in Pakistan, from February 2005 to January 2006, and the study by Hedayati et al. (37) was performed in Iran from June 2012 to June 2013. According to the same geographical area of both studies and also that the second study was about 6 years later, we can assume developing a new emerging antibiotic resistance among the S. aureus species in this area, but due to the small sample size, our evidence has not enough strength to prove it. Another assumption can be related to the microbiological tests that in the study by Hedayati et al. (37), samples were retrieved from the corneal scraping culture, but Rahim et al. (31) studied the germs isolated from the conjunctiva. Also, this resistance may be related to the purpose of the contact lens (cosmetic or therapeutic); as Hedayati et al. (37) mentioned, cosmetic contact lens users had less education about contact lens hygiene compared to patients who wore therapeutic contact lenses, but Rahim et al. (31) did not mention the number of users in each category.

Gentamicin has been studied by Lai et al. (27), Moriyama et al. (22), and Hoddenbach et al. (17), and they proved that *P. aeruginosa* is completely sensitive to it, but conversely in the studies conducted by Hedayati et al. (37) and Rasoulinejad et al. (39), *P. aeruginosa* was 100% resistant to this antibiotic. This paradoxical result firstly can be interpreted by the region in which the study is conducted. Hedayati et al. (37) and Rasoulinejad et al. (39) performed their studies in Iran, but studies by Lai et al. (27) and Moriyama et al. (22) and Hoddenbach et al. (17) were conducted respectively in China, Brazil, and the Netherlands. Thus, it can be concluded that in the Middle East especially Iran, P. aeruginosa species have developed antibiotic resistance to gentamicin. According to the date of studies, although Moriyama et al. (22) and Hoddenbach et al. (17) studied this issue between 2003 to 2009, Lai et al. (27) performed the study in about the same time as Hedayati et al. (37) and Rasoulinejad et al. (39) did between 2010 to 2013; therefore, this resistance is probably regional, and at least when the studies were conducted, the resistance was not spread to other regions. Most of these studies used the corneal culture method for identifying the bacteria, so we cannot attribute it to the sampling method. We should notice that there was another study in this area, Iran, which is conducted by Mohammadpour et al. (38) between 2009 to 2010 who studied on 52 patients with Pseudomonas keratitis exclusively and declared that Pseudomonas is 93% sensitive to gentamicin. This controversy between these three studies which were all held in Iran is doubtful, and antibiotic resistance mutation occurrence in about a 2-year interval seems impossible. This issue needs more investigations, and this resistance in Iran and probably surrounding countries should be considered in the treatment of *P. aeruginosa* keratitis and gentamicin prescription for bacterial keratitis should be avoided.

Konda et al. (15) declared that Pseudomonas spp., Serratia spp., and CoNS are most resistant against chloramphenicol, while Faghri et al. (30) demonstrated that these germs had 94.7% sensitivity to chloramphenicol. Konda et al. (15) performed the study on 125 patients from January 2001 to November 2011 in India, but Faghri et al. (30) held it on 77 patients in Iran, from January 2013 to August 2013. Both studies are prospective with a notable sample size and both used corneal cultures, but Konda et al. (15) also used contact lens and contact lens case cultures. One of the reasons for this meaningful difference in the sensitivity profile of chloramphenicol could be attributed to the dominant organism in the study by Konda et al. (15), which was Pseudomonas spp. (73.5%), However, in the study by Faghri et al. (30), the dominant organism was coagulase-negative Staphylococcus (49.3%), and Pseudomonas was only responsible for 7% of the cases. Thus, it can change the overall response to the treatment. We should notice that Konda et al. (15) did not report the percent of resistance to chloramphenicol, and Faghri et al. (30) calculated the overall sensitivity of microorganisms to chloramphenicol. However, the proximity of Iran and India and the short time interval between these two studies, as well as the remarkable sample size of these studies, make this meaningful difference more doubtful!

Karaca et al. (23) and Green et al. (29) reported that *P. aeruginosa* was 100% sensitive to vancomycin, but Mohammadpour et al. (38) mentioned complete resistance to vancomycin. Mohammadpour et al. (38) performed their study on 52 patients with exclusively Pseudomonas bacterial keratitis in Iran from March 2009 to March 2010 and used smears for gram staining and then cultured the specimens. Karaca et al. (23) studied 62 patients in Turkey from 2002 to 2018 and used corneal, contact lens, and storage case cultures. Green et al. (29) made an investigation in Australia, from January 1999 to December 2015, with 372 episodes of CLMK that were proved by corneal scraping cultures. These studies had non-different methods for isolation and identification of the organisms, and these differences cannot be attributed to the methodological issues. Also, as mentioned previously, the type of contact lens (therapeutic or cosmetic) could be an essential factor. In the study by Mohammadpour et al. (38), only 14% of the patients had therapeutic contact lenses. However, in the study by Karaca et al. (23), 85.5% of the patients had therapeutic contact lenses, and we previously mentioned that low education level in the cosmetic contact lens users might be related to the consequent outcomes. It also can be attributed to the different subspecies with various vancomycin genetic resistances, but according to the proximity of Iran and Turkey, this meaningful difference needs to be investigated more in the future.

While most of the similar articles were narrative review studies, we considered all of the related articles over time as a systematic review in this article. One of the strengths of the present study is that it reveals the controversies among different studies and, according to the standard and rational events, suggests some assumptions or logical reasons. On the other hand, there is an agreement in most of the details between this article and other previous similar systematic review studies that were written by Willcox (42), Mattila et al. (43), Eggink et al. (44), and Zimmerman et al. (45). There has been a consensus on this issue in which Pseudomonas is the most common bacterial organism in microbial keratitis, and the fluoroquinolone family, especially ciprofloxacin, is the first-line treatment. Another essential strength point of this study is its recency; the last review study in this issue was performed by Willcox (42) in 2012, and in the last 9 years, there were no similar studies. Most of the previous studies did not focus on bacterial keratitis exclusively and reviewed Acanthamoeba keratitis alone or along with bacterial keratitis. They also usually discussed other risk factors rather than focusing on contact lens-induced keratitis, and sometimes they approached the risk factors, epidemiologic factors, and other issues but did not focus on antibiotic treatment and antibiotic sensitivity profile. However, in this article, we discussed that bacterial keratitis only related to contact lens wear and its antibiotic treatment and antibiotic sensitivity profile.

There were also limitations in this study. Some of the articles which were selected for the review were written in a language other than English, e.g., Chinese, and we could not use them for review. On the other hand, the total number of articles that met the inclusion criteria was limited. In reviewing the treatment approaches, we did not consider combination therapy, while some studies like those of Willcox (42) and Zimmerman et al. (45) paid great attention to combination therapy instead of monotherapy. Corticosteroid therapy is also considered in the study of Zimmerman et al. (45) as an important part of treatment, unlike our study.

### Suggestions

Around the world, Pseudomonas is sensitive and responds well to gentamicin, but in the Iran region, in different studies, converse results have been observed (37, 39).

*S. aureus* was sensitive to ciprofloxacin, but in the study conducted by Hedayati et al. (24), all three S. aureus samples were resistant to it.

As a broad-spectrum antibiotic, vancomycin is a potent antibiotic that most germs respond to and is prescribed empirically (23, 29). It has been proven in various studies that vancomycin is an effective anti-Pseudomonas agent. However, the existence of 100% resistance to this antibiotic in a study (38) with a considerable sample size can be indicative of a developing resistance among the bacteria in the Middle East, and because of the broad use of this antibiotic in hospital settings, developing resistance is an important and serious issue.

In addition, although resistance to chloramphenicol has been proven in most studies, in a study conducted in Iran (30), a significant sensitivity was reported.

Almost all of these converse results were detected in the Middle East region, especially Iran and Pakistan, so more investigation is needed to reveal the underlying reason for these events. We suggest future *in vitro* molecular studies to identify different subspecies, developing antibiotic resistance and genetic mutations in this regard.

Sampling from various places such as conjunctiva or corneal scrapings may significantly affect the final result of the sensitivity profile test in the reviewed articles. Consequently, different sources of isolated bacteria may lead to different antibiotic sensitivity profiles even in the same geographical areas. In this regard, it is suggested to evaluate the effect of sampling from different places in future studies.

For systemic Pseudomonas infections in nosocomial settings, piperacillin/tazobactam as an anti-Pseudomonas antibiotic is commonly used (46). Also, for the treatment of non-contact lens-related keratitis, topical piperacillin/tazobactam is used in resistant keratitis cases as an effective antibiotic and has promising outcomes (47). Therefore, for future studies, the response to treatment with this antibiotic in the severe resistant cases of contact lens-related bacterial keratitis can be evaluated.

## Conclusion

Among the reviewed articles, *Pseudomonas aeruginosa* was the most common bacteria, and Staphylococcus spp. such as S. aureus and Coagulase-negative spp. were the second, and Serratia marcescens was the third one. Commonly isolated bacteria were most sensitive to fluoroquinolones and aminoglycosides, especially ciprofloxacin and gentamicin, respectively, and most resistant against penicillin, cephalosporins, and chloramphenicol. Almost all patients responded well to antibiotic therapy, and some untreated cases needed further surgical interventions. In most of the reviewed studies, bacteria were susceptible to gentamicin and vancomycin and fully resistant against chloramphenicol. However, in the Middle East region, especially Iran, there were some different results about these antibiotics, which need more *in vitro* and clinical studies about the sensitivity and resistance of germs against them.

## Data Availability Statement

The original contributions presented in the study are included in the article/supplementary material. Further inquiries can be directed to the corresponding authors.

## Author Contributions

HH, MZ, MN: designed the study. MZ, AG, AE, SG: performed the search, study selection, and data synthesis. HH, MZ, AG, AE, SG: wrote the first draft of the manuscript. MN, MZ, HH: revised the article. All authors contributed to the article and approved the submitted version.

## Conflict of Interest

The authors declare that the research was conducted in the absence of any commercial or financial relationships that could be construed as a potential conflict of interest.

## Publisher’s Note

All claims expressed in this article are solely those of the authors and do not necessarily represent those of their affiliated organizations, or those of the publisher, the editors and the reviewers. Any product that may be evaluated in this article, or claim that may be made by its manufacturer, is not guaranteed or endorsed by the publisher.
